# Weighted gene co-expression network analysis to identify key modules and hub genes associated with paucigranulocytic asthma

**DOI:** 10.1186/s12890-021-01711-3

**Published:** 2021-11-02

**Authors:** Min Li, Wenye Zhu, Chu Wang, Yuanyuan Zheng, Shibo Sun, Yan Fang, Zhuang Luo

**Affiliations:** 1 Department of Respiratory and Critical Care Medicine, West China Hospital, Sichuan University, Chengdu, The People’s Republic of China; 2 Department of Respiratory and Critical Care Medicine, First Affiliated Hospital of Kunming Medical University, Kunming, The People’s Republic of China; 3 Department of Pharmacy, First Affiliated Hospital of Kunming Medical University, Kunming, The People’s Republic of China

**Keywords:** Paucigranulocytic asthma, Immune status, WGCNA, Hub genes

## Abstract

**Background:**

Asthma is a heterogeneous disease that can be divided into four inflammatory phenotypes: eosinophilic asthma (EA), neutrophilic asthma (NA), mixed granulocytic asthma (MGA), and paucigranulocytic asthma (PGA). While research has mainly focused on EA and NA, the understanding of PGA is limited. In this study, we aimed to identify underlying mechanisms and hub genes of PGA.

**Methods:**

Based on the dataset from Gene Expression Omnibus(GEO), weighted gene coexpression network analysis (WGCNA), differentially expressed genes (DEGs) analysis and protein–protein interaction (PPI) network analysis were conducted to construct a gene network and to identify key gene modules and hub genes. Functional enrichment analyses were performed to investigate the biological process, pathways and immune status of PGA. The hub genes were validated in a separate dataset.

**Results:**

Compared to non-PGA, PGA had a different gene expression pattern, in which 449 genes were differentially expressed. One gene module significantly associated with PGA was identified. Intersection between the differentially expressed genes (DEGs) and the genes from the module that were most relevant to PGA were mainly enriched in inflammation and immune response regulation. The single sample Gene Set Enrichment Analysis (ssGSEA) suggested a decreased immune infiltration and function in PGA. Finally six hub genes of PGA were identified, including *ADCY2*, *CXCL1*, *FPRL1*, *GPR109B, GPR109A* and *ADCY3,* which were validated in a separate dataset of GSE137268.

**Conclusions:**

Our study characterized distinct gene expression patterns, biological processes and immune status of PGA and identified hub genes, which may improve the understanding of underlying mechanism and provide potential therapeutic targets for PGA.

**Supplementary Information:**

The online version contains supplementary material available at 10.1186/s12890-021-01711-3.

## Background

Asthma is a heterogeneous disease with different phenotypes that vary in natural history, severity of the disease and response to anti-inflammatory therapy [1]. According to the airway inflammation subtypes, asthma can be categorized into four distinct inflammatory phenotypes: eosinophilic asthma (EA), neutrophilic asthma (NA), mixed granulocytic asthma (MGA), and paucigranulocytic asthma (PGA) [2]. Recently, extensive attentions have been paid to EA and NA, which have been successfully applied to clinical research and asthma management. For instance, airway eosinophilic inflammation is somewhat related to atopy and EA has a good response to inhaled corticosteroids (ICS) [3–6]. While airway neutrophilic inflammation is associated with the exposure to environmental pollutants (such as smoking) or the presence of bacterial or viral infection [7]. Additional therapy of macrolide may be more suitable for NA with respect to reducing airway neutrophilic inflammation [8].

However, as one of the most common phenotypes of asthma, PGA are still poorly understood and researches on PGA are limited [9]. Some studies considered PGA to be a special phenotype driven by macrophages or mast cells other than eosinophils or neutrophils [10, 11]. Other studies suggested that PGA may represent a non-inflammatory type or a phenotype with a low grade of eosinophilic inflammation [12]. The precise characteristics and pathobiology of PGA are not well delineated. It is urgent to unveil inflammatory and immune mechanisms underlying PGA.

The rapid development of microarray and high-throughput sequencing technologies facilitate the study of asthma in genetic level. An earlier study conducted a hierarchical cluster analysis based on the transcriptional profiles of asthma and identified three clusters that showed similarities with the inflammatory phenotypes of EA, NA and PGA [10]. However, there are no studies that specifically address the transcriptional features of PGA. The key gene modules or hub genes of PGA are still unknown. Traditional methods rely on differential expression detection to identify potential biomarkers or targets, but may miss useful genes. Weighted gene coexpression network analysis (WGCNA) is a bioinformatic method to explore complex interactions among gene expression profiles. According to expression similarity, WGCNA can transform gene expression data into potentially biologically relevant modules and reveal relationships between the gene modules and external clinical traits by using an intramodular hub gene or module eigengene [13]. It is quite helpful in identifying hub genes or therapeutic targets.

In this study, we sought to identify the hub genes located in the regulatory center of PGA using WGCNA and other bioinformatic methods. Additional biological functional analyses were also conducted to investigate the biological processes, related pathways and immune status of PGA. The results will help to shed light on hidden mechanisms and identify therapeutic targets of PGA.

## Methods

### Data collection

Microarray RNA expression dataset of GSE45111 was downloaded from the Gene Expression Omnibus (GEO; https://www.ncbi.nlm.nih.gov/geo/). It was generated from samples of induced sputum in 47 asthma patients. Adults with stable asthma were recruited and those who with recent (past month) respiratory tract infection, asthma exacerbation, unstable asthma, change in therapy and current smoking were excluded. All the patients in the dataset received ICS therapy and they were grouped according to the inflammatory phenotypes using sputum cell counts. Patients with a sputum proportion of < 61% neutrophils and < 2% eosinophils were classified as PGA, with ≥ 61% neutrophils and < 2% eosinophils classified as NA, with < 61% neutrophils and ≥ 3% eosinophils classified as EA, and with ≥ 61% neutrophils and ≥ 3% eosinophils classified as MGA, respectively [2, 14]. All the asthmatics other than PGA were defined as non-PGA in our study. The data was log-transformed, normalized and baseline-converted to the median of all samples. The dataset was based on the platform *GPL6104* (Illumina human Ref-8 v2.0 expression beadchip, Illumina, Inc., San Diego, California, USA).

### Weighted gene co-expression network analysis

The gene expression matrix from GSE45111 were used to perform weighted gene co-expression network analysis (WGCNA). The adjacency matrix was transformed into a topological overlap matrix (TOM) to estimate the distance between each gene pair. And then hierarchical clustering with the average and dynamic methods were employed to build the cluster tree and to classify the genes into different modules. The modules that were most relevant to the paucigranulocytic airway inflammation were selected for subsequent analysis. The soft-thresholding power β was calculated in the construction of each module using the pickSoftThreshold function of WGCNA, which provides a suitable power value for network construction by calculating the scale-free topology fit index for a set of candidate powers that range from 1 to 20. In this study, a suitable soft threshold of seven was selected, as it met the degree of independence of 0.85 with the minimum power value (R^2^ = 0.851). Then the WGCNA algorithm implemented in the R package was used to identify the co-expression gene modules. The minimum number of genes for each module was set to 50. The strength of the interactions between modules was analyzed and visualized by a heatmap. “WGCNA” R package was used to perform the analysis.

### DEG analysis and interactions with the modules of WGCNA

DEGs between PGA and non-PGA were screened with a threshold of a |fold change (FC)|> 1.5 and *adjp*-value (false discovery rate, FDR) < 0.05. Then intersection of DEGs and the genes in the modules that were most relevant to PGA were taken. DEGs were screened and visualized by the R package of “limma” and “ggplot2” [15, 16]. “VennDiagram” were applied to perform the intersection analysis [17].

### Biological function and pathway enrichment analysis

Using the intersection of the DEGs and WGCNA, we conducted GO (Gene Ontology) and KEGG (Kyoto Encyclopedia of Genes and Genomes) enrichment analyses by "clusterProfiler" R package [18–20]. The analyses were based on a corrected Fisher`s exact test. A *p* value < 0.05 was considered statistically significant. The results were visualized using “ggplot” R packages.

### Immune infiltration analysis

Based on the above-mentioned intersection of the genes, infiltration of immune cell and related pathway or function were quantified by ssGSEA, which calculated an enrichment score that represents the immune cell infiltration level and activity of immune related pathways [21]. Mann–Whitney test with *p *values adjusted by Benjamini and Hochberg (BH) correction were used to compare the ssGSEA scores between the two clusters. R package of “gsva” was used to conduct the analysis. The annotated gene set and definition of each immune term are based on the study by Liang et al. [22].

### Protein–protein interaction network

The intersection of the DEGs and WGCNA were imported to STRING (version 11.0) database (http://string-db.org) to perform protein–protein interaction (PPI) network analysis [23]. The active interaction sources included text mining, experiments, databases, co-expression, neighborhood, gene fusion and co-occurrence. The minimum score required for the interaction was set at the highest level of confidence (0.90). To screen the hub genes of PGA, topological analysis of Maximal Clique Centrality (MCC), Edge Percolated Component (EPC), Maximum Neighborhood Component (MNC), Closeness and Radiality were applied [22]. The intersection of the top ten genes with highest scores individually calculated by each of the five algorithms were finally selected as hub genes. Cytoscape version 3.4.0 and Cytohubba plugin were used for network visualization and hub gene identification [24, 25].

### Validation of the hub genes

The expression profiles of the hub genes were validated in a separate dataset of GSE137268. The dataset contained gene expression profiles of the induced sputum from 16 PGA and 38 non-PGA patients. The data was log-transformed, normalized and baseline-converted to the median of all samples. Like the GSE45111, the dataset was also based on the platform *GPL6104* (Illumina human Ref-8 v2.0 expression beadchip, Illumina, Inc., San Diego, California, USA). The expression profile of the microarray dataset was analyzed using the same methods as aforementioned for GSE45111. In addition, receiver operating characteristic (ROC) curve analysis was conducted for each hub gene. The area under the curve (AUC) was used to evaluate the diagnostic accuracy of the six hub genes for PGA.

## Results

**Fig. 1:**
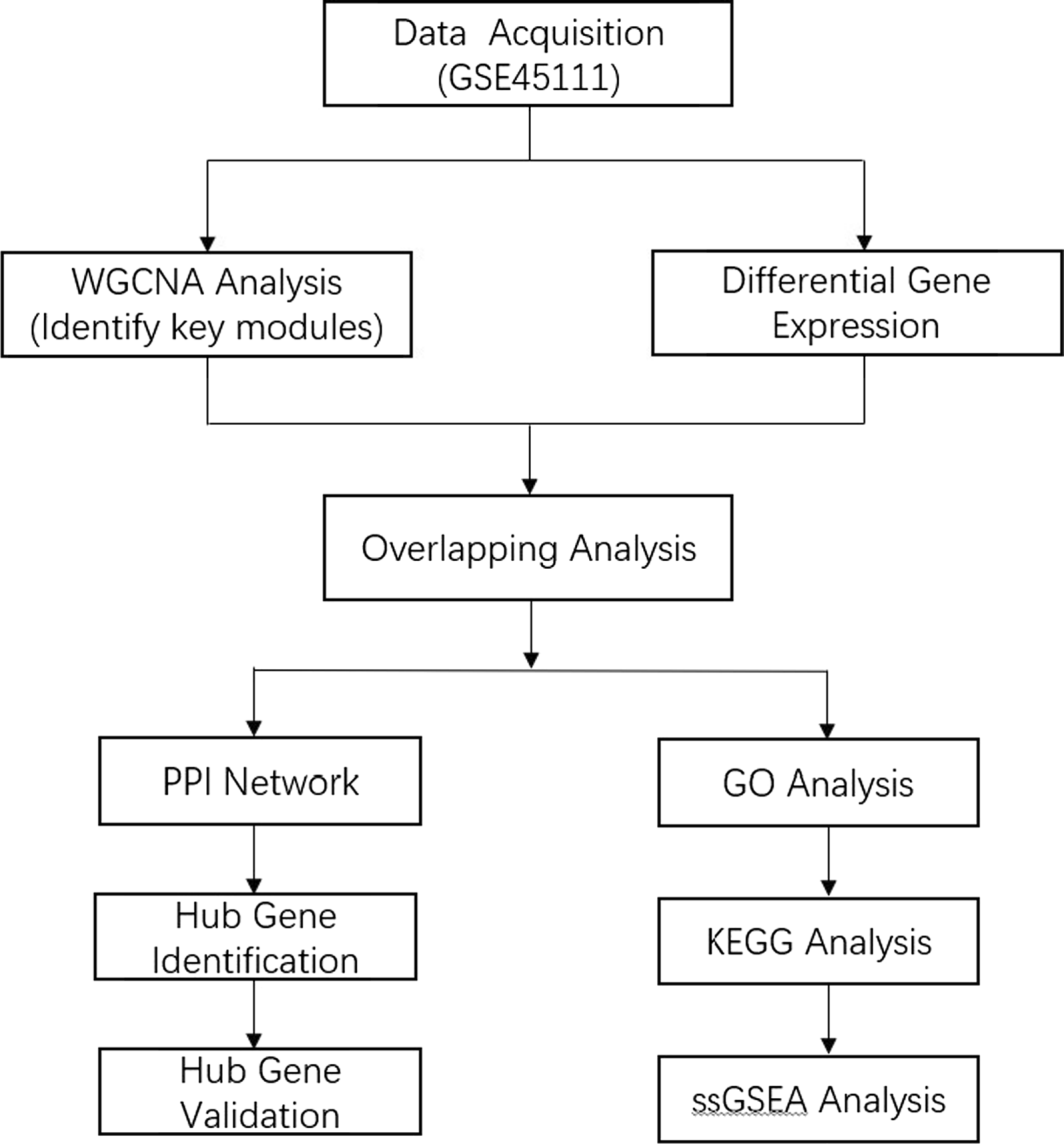
Flowchart of the bioinformatics methods used in the present study; DEGs, differentially expressed genes; WGCNA, weighted gene co-expression network analysis; GO, gene ontology; KEGG, Kyoto encyclopedia of genes and genomes enrichment analysis; PPI, protein–protein interaction; ssGSEA, single-sample gene set enrichment analysis

In total, 47 samples (18 PGA and 29 non-PGA) from GSE45111 were used to perform WGCNA, DEGs analysis, functional enrichment analysis and PPI analysis. 54 samples from GSE137268 were used to validate the identified hub genes. The demographic and clinical data of patients in the two datasets were summarized in Additional file 1: Table S1. The flowchart of the study is presented in Fig. 1.

### Co‑expression network construction and PGA‑specific module identification

**Fig. 2:**
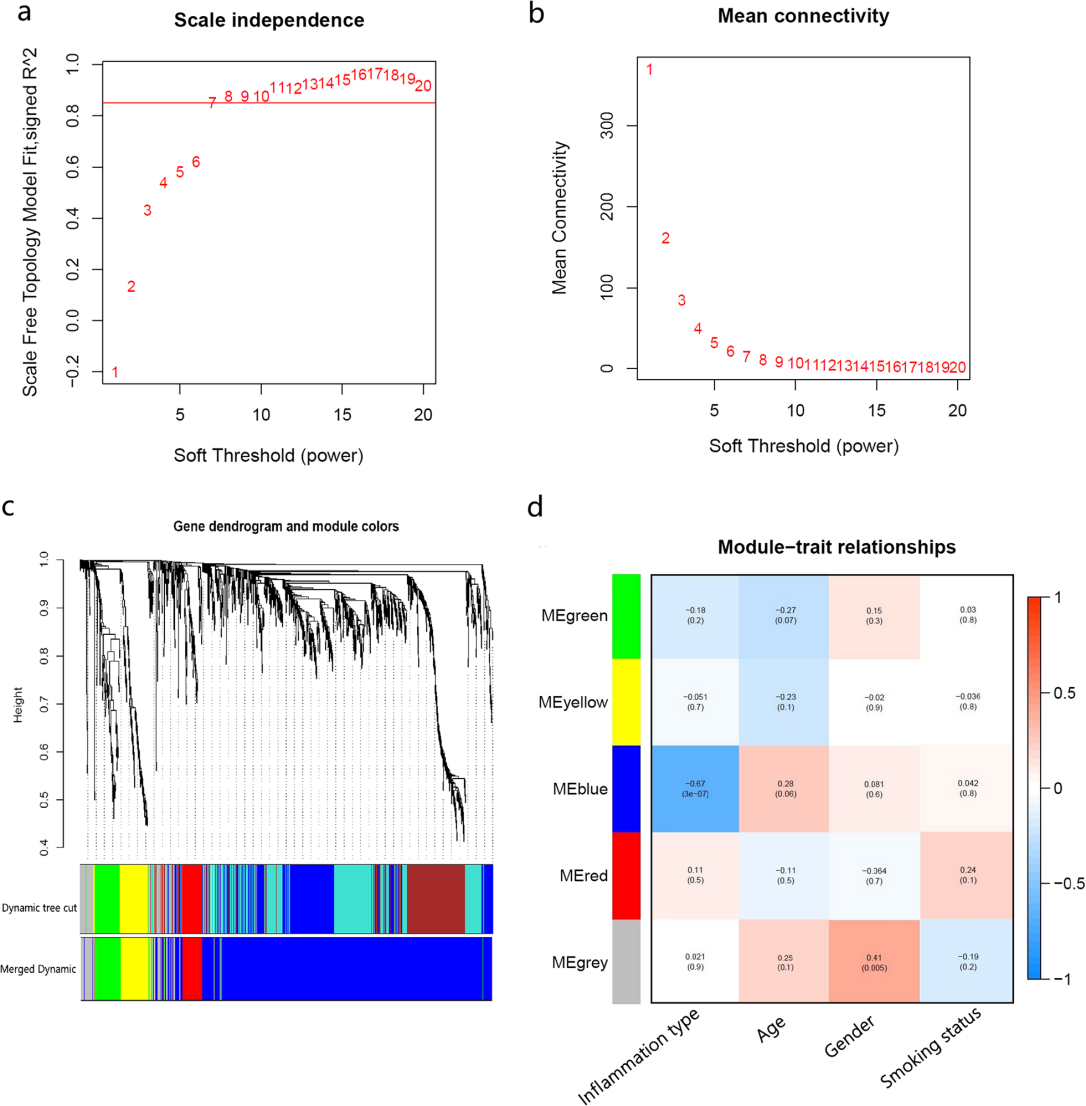
Construction of weighted co-expression network. **a**, **b** Analysis of network topology for a set of soft‐thresholding powers. **c** Cluster dendrogram. Each color represents one specific co-expression module, and branches above represent genes. **d** Heatmap of the correlation between modules and clinical characteristics including airway inflammation type (paucigranulocytic inflammation or non-paucigranulocytic inflammation), age, gender and smoking status. Each cell contains the correlation coefficient which correspond to the cell color: red represents positive correlation and blue represents negative correlation. *p* values are stated in the brackets

The expression profiles of 18,170 genes were used to conduct WGCNA. Hierarchical cluster analysis of these samples was then performed with the flashClust function, and the results are shown in Additional file 1: Figure S1. A soft‑threshold of 7 was chosen to obtain the approximate scale‑free topology with a scale‑free topology fit index > 0.85 (R^2^ = 0.851) and the lowest power (Fig. 2a,b). Using a dynamic tree-cutting algorithm (0.25 as the merging threshold), five modules were finally identified (Fig. 2c). Of these, the blue model containing 930 genes was significantly associated with paucigranulocytic airway inflammation (r = −0.67, *p* = 3e−7) (Fig. 2d), which let us to select blue modules for next analysis. No modules were found to be correlated with age, gender or smoking status.

### DEG analysis and interactions with the PGA‑specific module

**Fig. 3:**
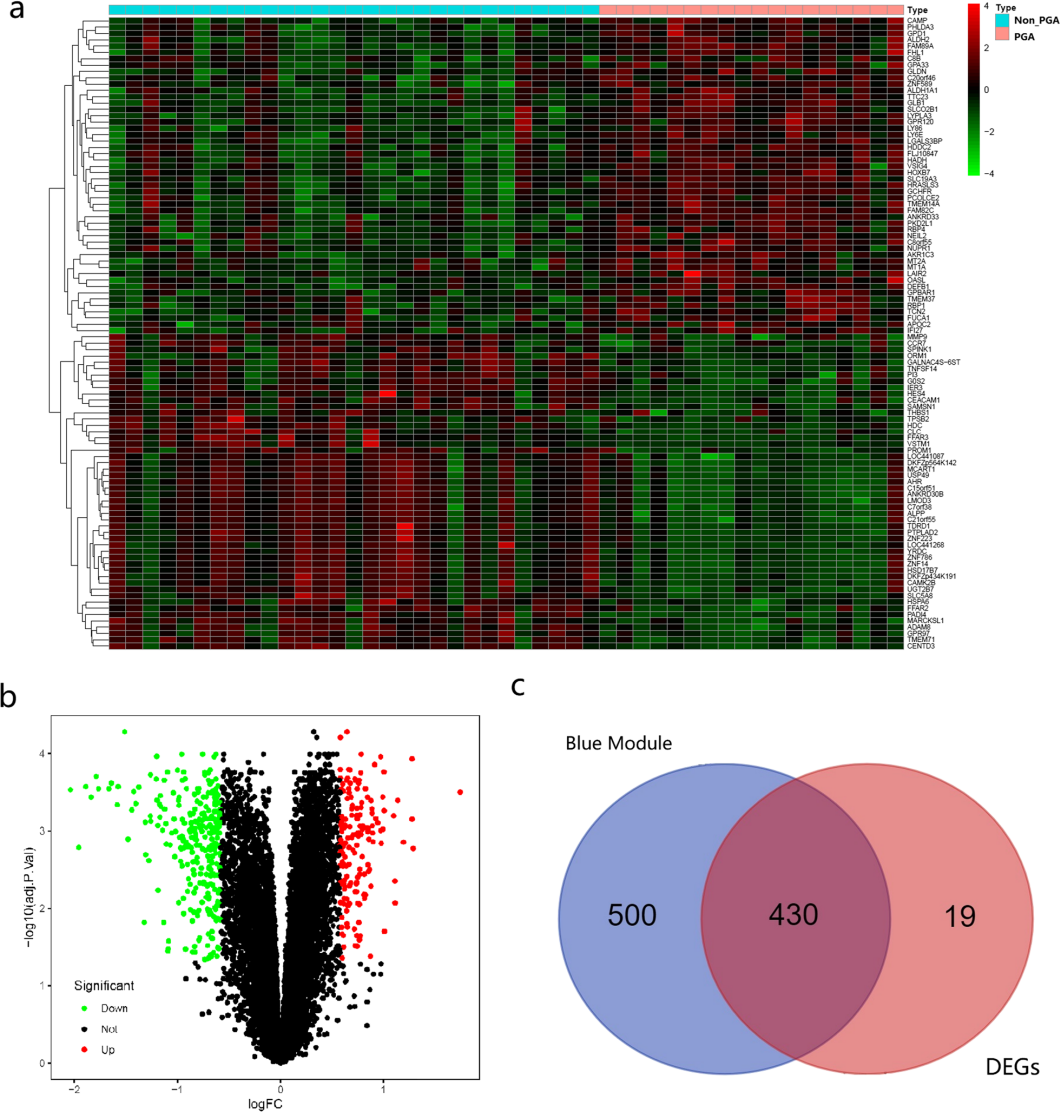
**a** The heatmap of the top 100 DEGs. The red color indicates the higher gene expression value while the green color indicates the lower gene expression. **b** Volcano plot of DEGs. Green indicates downregulated genes, and red indicates upregulated genes. **c** Intersection of DEGs and the genes from blue module

DEG analysis between the PGA and non-PGA suggested that a total of 449 DEGs (161 up-regulated genes and 288 down-regulated genes) were identified with the threshold of |FC|> 1.5 and FDR < 0.05. The heatmap and volcano plot of the DEGs are shown in Fig. 3a and b. The Venn diagram (Fig. 3c) exhibited a notable overlap between the DEGs and the genes in the blue module. The intersection of the DEGs and genes in this module were used for further analysis (430 genes).

### Functional analyses of the overlapped genes

**Fig. 4:**
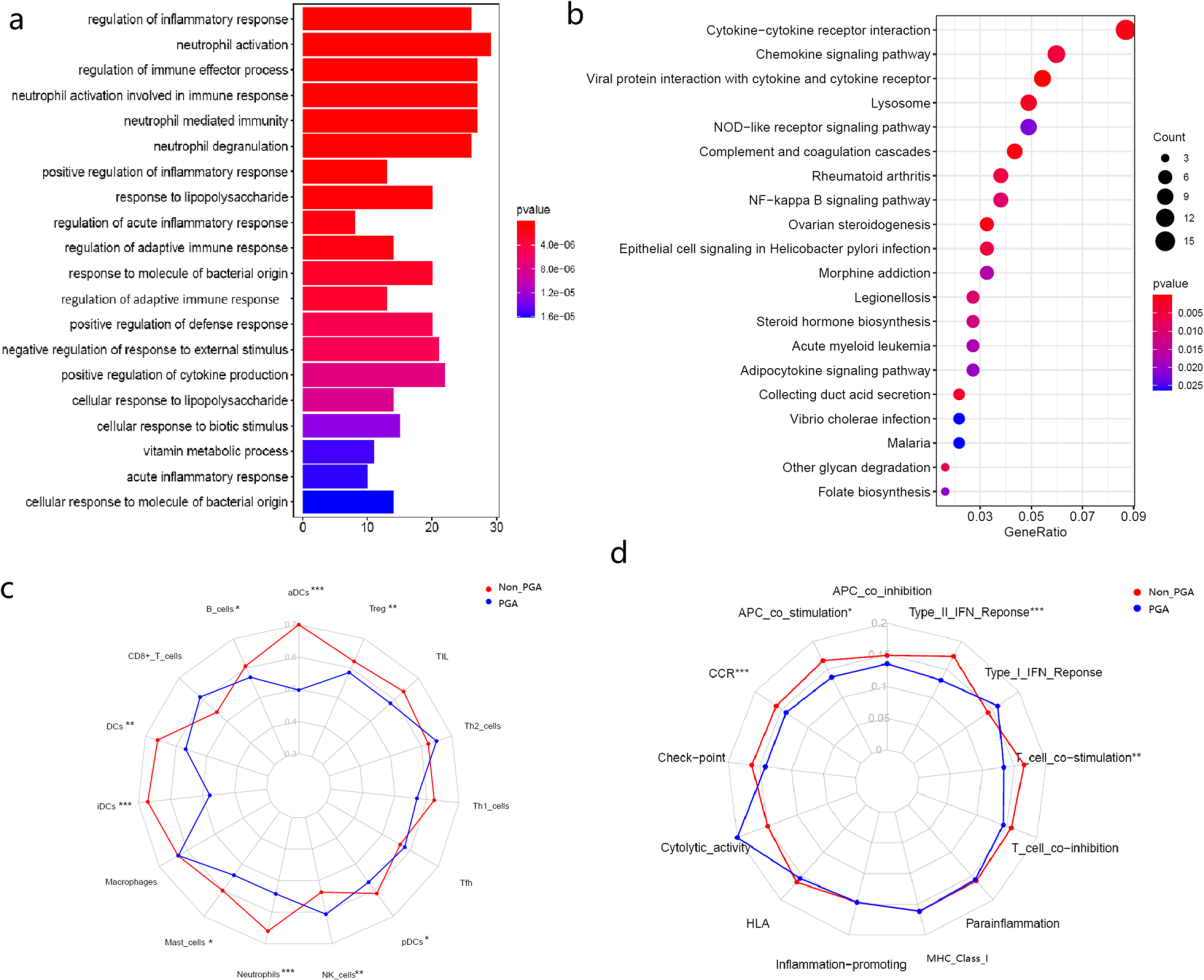
**a** Representative results of GO enrichment in biological process terms. **b** Representative results of KEGG pathway analysis. **c** The ssGSEA score of 15 immune cells. **d** The ssGSEA score of 13 immune related functions or pathways. *p *values were presented as: *p < 0.05; **p < 0.01; ***p < 0.001

Based on the intersection of the DEGs and WGCNA, GO and KEGG enrichment analyses were performed. Go analysis suggested that items highly related to the regulation of immune and inflammatory response, such as regulation of inflammatory response (GO:0050727), regulation of immune effector process (GO:0002697) and regulation of adaptive immune response (GO:0002819), were significantly enriched. The KEGG pathway enrichment analysis suggested that several items related to signal transduction, like cytokine-cytokine receptor interaction (hsa04060), NF-kappa B signaling pathway (hsa04064), NOD-like receptor signaling pathway (hsa04621) and chemokine signaling pathway (hsa04062), were significantly enriched (Fig. 4a, b).

The ssGSEA indicated different immune status between the PGA and non-PGA. As shown in Fig. 4c and d, compared with non-PGA, PGA had a lower immune infiltration score in majority of the immune cells, such as dendritic cells (DCs), B cells, mast cells, neutrophils, NK cells and Treg (All *p* < 0.05). Meanwhile, the immune scores in the immune function were also tend to be lower, including antigen presentation process (APC) co-stimulation, CCR (chemokine receptors), T-cell co-stimulation and Type II IFN response (All *p* < 0.05).

### Construction of PPI network and hub gene analysis

**Fig. 5:**
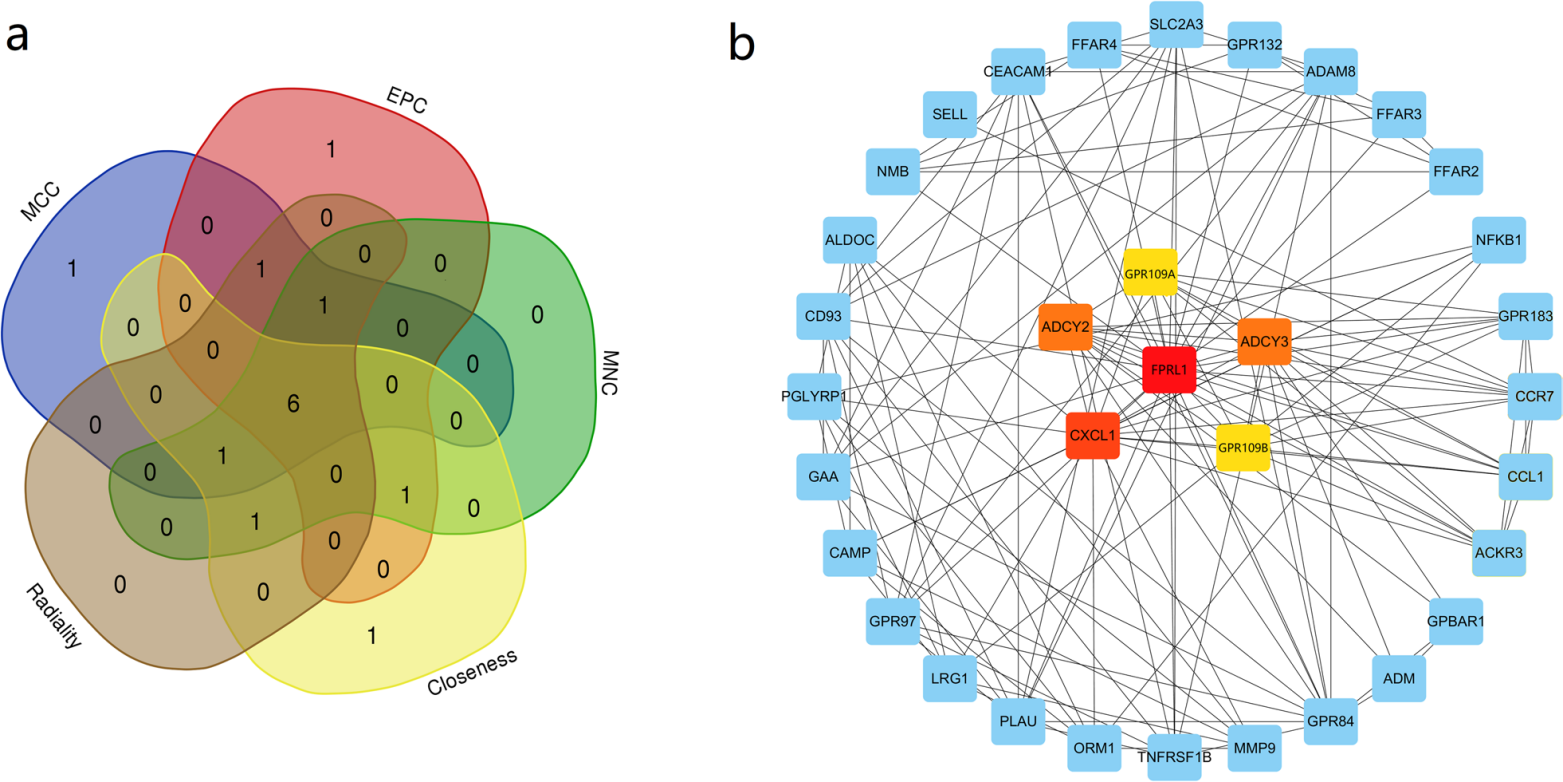
Identification of hub genes. **a** Venn diagram demonstrates the intersection of the top ten genes with highest scores individually calculated by each of the five algorithms (MCC, EPC, MNC, Closeness and Radiality); **b** the visualization of the six hub genes and their adjacent genes

430 overlapped genes were imported into STRING to develop a PPI network (Additional file 1: Figure S2). Based on the intersection of the top ten genes with highest scores individually calculated by each of the five algorithms (MCC, EPC, MNC, Closeness and Radiality), six genes were finally identified as the hub genes of PGA, which included: *FPRL1, CXCL1, ADCY2, ADCY3, GPR109A* and *GPR109B* (Fig. 5).

### Validation of the hub genes

**Fig. 6:**
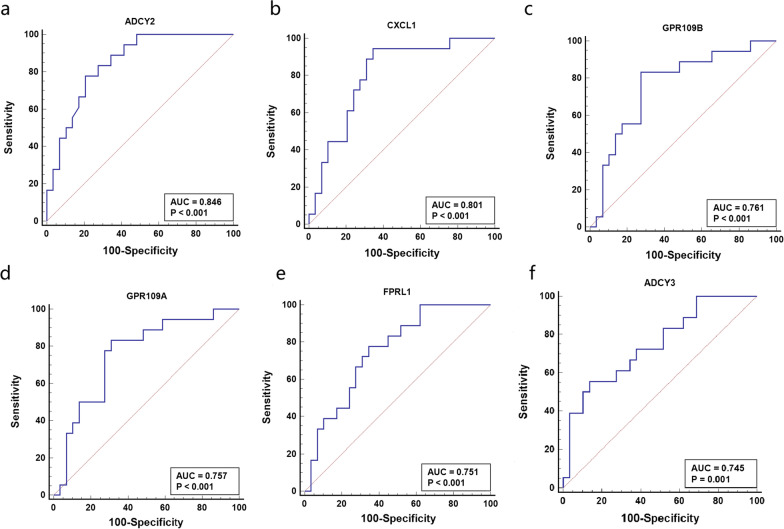
The ROC analysis of the hub genes for PGA

The differential expressions of the six hub genes between the PGA and non-PGA were validated in the GSE137268. The results suggested that the expression patterns of the hub genes in GSE137268 were almost similar to GSE45111. The expression level of *ADCY3* was up-regulated while the remaining five hub genes were down-regulated in the PGA. The fold change of the hub genes between the PGA and non-PGA were also similar in the two datasets (Additional file 1: Table S2, S3). ROC curve analysis suggested that the AUC for *ADCY2* was 0.85 (*p* < 0.001), followed by *CXCL1, GPR109B, GPR109A, FPRL1,* and *ADCY3.* The hub genes indicated a moderate discrimination ability between PGA and non-PGA (Fig. 6).

## Discussion

As one of the most common phenotypes of asthma, PGA accounts for the 31–51.7% of asthma [2, 26, 27]. However, unlike EA or NA, researches on PGA are limited and its characteristics have not been well delineated [9]. To the best of our knowledge, this is the first transcriptomics study on PGA to identify key gene modules and hub genes. In the present study, we investigated the transcriptome of 18 asthmatic patients with a phenotype of PGA and 29 controls of non-PGA. Using integrated analyses of DEGs, WGCNA and PPI, we identified and validated six hub genes of PGA, including *ADCY2, CXCL1, FPRL1, GPR109B, GPR109A* and *ADCY3*. In comparison with strategies focused on individual gene, network-based methods are more suitable to reveal global biological activity. WGCNA focuses on the correlations between the co-expression modules and the external clinical traits, not merely the differences in gene expression patterns, and thus the results are more reasonable [13]. Consequently, the analysis allows the identification of candidate genes and the modules potentially linked to the biological function of interest. The GO, KEGG and ssGSEA analyses were further performed to elucidate the potential biological process, pathways and immune functions that may be implicated in the pathogenesis of PGA. These results may enhance the current understanding of the mechanisms underlying PGA and provide potential therapeutic targets for newly developed treatments.

It has been previously reported that PGA most likely represents a “benign” phenotype of asthma and it is associated with a good response to anti-asthma treatment. Several studies have suggested that PGA has distinct inflammation features compared with EA or NEA [26, 28, 29]. In the enrichment analysis of our study, GO terms related to the inflammation response and immune regulation were significantly enriched, such as regulation of inflammatory response (GO:0050727), regulation of immune effector process (GO:0002697) and regulation of adaptive immune response (GO:0002819), indicating that the inflammatory and immunological characteristics were different between PGA and non-PGA. Ntontsi et al. found that patients with PGA express lower levels of inflammatory biomarkers in exhaled air and induced sputum supernatants compared with other inflammatory phenotypes, representing a less intense inflammatory process [26]. Demarche et al. also showed that PGA may display a low-grade airway inflammation [12]. The results of ssGSEA in our study showed that the scores of immune cell infiltration and immune functions were lower in PGA than non-PGA, which seems to support that PGA represents a less intense immune response and the viewpoint that PGA is somewhat a kind of phenotype with low degree of inflammation [12]. According to Ntontsi et al. in some patients with PGA, the “absence of inflammatory response” could possibly be the results of a pre-existing eosinophilic asthma adequately treated with ICS in which there is no neutrophilic inflammation. In other words, some PGA patients may be the result of the successful therapeutic intervention. The hypothesis may partly explain the low degree of immune response presented in PGA and its good response to anti-asthma treatment [26]. However, Deng et al. found that the phenotype of PGA was stable and that most patients with PGA had not undergo an inflammatory phenotype transition. Their study did not support the hypothesis that all subjects with PGA represent a cross sectional view related to disease activity or represent a treatment success. Instead, it indicated that most patients with PGA could constitute an independent phenotype [30]. More studies are required to address these concerns. Meanwhile, it should be noted that the immune scores of most immune cells were lower in PGA except for NK cell. Although the mechanisms of NK cells in the regulation of inflammation of asthma are not fully elucidated, recent studies have suggested that NK cells in asthma inflammation can be protagonistic or antagonistic, depending on the environmental agent that is used to elicit the disease (allergen, diesel exhaust particles and virus) and the phase of the disease (the sensitization phase, the effector phase and the resolution phase) [31–34]. Therefore many factors, including the type of the environmental trigger, the phase of inflammation and the cytokine milieu between PGA and non-PGA, should be further investigated.

Our study suggested the different gene expression patterns between PGA and non-PGA. Six hub genes were identified based on the combination analyses of DEGs, WGCNA and PPI. Of these, *ADCY3* was up-regulated in PGA, while the remaining five hub genes were down-regulated. The expression patterns were further validated in a separate dataset (GSE137268). The majority of the hub genes were involved in the regulation of immune response and inflammation. For example, the up-regulation of *ADCY3* suggests an increase in cAMP formation, which could suppress inflammatory function in DCs [35]. *FPRL1* was reported to be implicated in several immune processes, such as chemotactic migration and the production of reactive oxygen species (ROS) [36]. Several agonistic and antagonistic peptide sequences for the *FPRL1* receptor have been investigated as drug candidates for inflammatory diseases including asthma [37]. The remaining hub genes were found to be involved in the migration of inflammatory cells. *GPR109B* participated in the migration of eosinophils to the sites of inflammation [38]. *CXCL1* was found to be a chemoattractant for neutrophil recruitment during tissue inflammation [39]. *GPR109A* was expressed in many immune cells, including macrophages, monocytes, neutrophils and DCs. Activation of *GPR109A* has been found to be implicated in several diseases where inflammation contributes to the underlying pathophysiology such as obesity, colitis and neurodegenerative disorders [40]. But its role in asthma is still not elucidated. The expression patterns of these genes that related to the immune cells activation or migration may explain the decreased ssGSEA score of immune status in PGA. Difference in chemotaxis and migration of the immune cells between PGA and non-PGA may be an important factor that leads to the different inflammatory characteristics of the two asthma phenotypes.

Our study was different in many respects from the original study for GSE45111 [41]. First, the objective of the study was to find gene signatures that could discriminate eosinophilic asthma from other phenotypes and to investigate its predicted value for ICS treatment response. In our study, we focused on identifying the hub genes for PGA. Gene signatures in the original study were identified based on the differential expression analysis, while in our study the hub genes were identified by the combination analyses of DEGs, WGCNA and PPI. We conducted more comprehensive bioinformatic analyses such as GO and KEGG enrichment analysis, ssGSEA, WGCNA and PPI analysis. These bioinformatic analyses were not performed in the original study.

It should be mentioned that although the identification of asthma inflammatory phenotype in both datasets used in our study was based on the cross-sectional data of induced sputum, asthma inflammatory phenotypes identified by this method are proved to be stable by many studies [30, 42–44]. Actually, induced sputum is currently the best available noninvasive assessment of bronchial inflammation in asthma, and it is regarded as the gold standard for asthma inflammatory phenotyping [45]. This method has been widely adopted in many of studies [46–48]. GINA guideline also recommends to use the method to confirm asthma inflammatory phenotype [49]*.* Deng et al. particularly focused on the PGA and found the phenotype of PGA identified by induced sputum was stable and that majority of the patients with PGA had not undergone an inflammatory phenotype transition after one-month fixed anti-asthma treatment with ICS [30]. In our study, the subjects in GSE45111 were stable asthmatics. Those who with recent (past month) respiratory tract infection, asthma exacerbation, unstable asthma, change in therapy and current smoking were excluded [39]. Taking account of all these factors into consideration, sputum phenotypes in the dataset of GSE45111 could be considered as stable.

The present study had several limitations. Firstly, we analyzed a single platform of a dataset and the sample size relatively was small, which may affect the stability of our study. Although we have validated our findings in a separate dataset, the results should be interpreted carefully. Besides, more sociodemographic characteristics and some other important clinical traits, such as pulmonary function or exacerbation history were absent in the original datasets, so we cannot perform a more comprehensive analysis. Finally, our study is based on a in silico analysis, more studies aimed at elucidating the further mechanisms of the identified hub genes in PGA are desired in the future.

## Conclusions

In summary, our study suggested that PGA and non-PGA were different in gene expression patterns, biological processes, related pathways and immune status. With comprehensive analyses of WGCNA and other related bioinformatic methods, we constructed a weighted co-expression network and identified a key gene module that associated with PGA. Finally, six hub genes of PGA were identified and then validated in a separate dataset. The different gene expression patterns, biological processes and immune status between PGA and non-PGA indicated the heterogeneity of asthma at level of molecular biology, suggesting the requirement of individualized treatment for different phenotypes of asthma. Our results may improve the understanding the heterogeneity of asthma and the underlying mechanism of PGA, providing potential therapeutic targets for patients with PGA.

## Supplementary Information

**Additional file 1**. **Table S1**: Baseline characteristics of the GSE45111 and GSE137268; **Table S2 and S3**; Validation of the hub genes; **Figure S1**: sample dendrogram and trait heatmap; **Figure S2**: Protein–protein interaction network analysis.

## Data Availability

The dataset analyzed in this study can be derived from public repositories: GSE45111dataset (https://www.ncbi.nlm.nih.gov/geo/query/acc.cgi?acc=GSE45111) and GSE137268 (https://www.ncbi.nlm.nih.gov/geo/query/acc.cgi?acc=GSE137268).

## Abbreviations

APC: Antigen presentation process
BP: Biological process
CCR: Chemokine receptors
CI: Confidential interval
DC: Dendritic cell
DEGs: Differentially expressed genes
EA: Eosinophilic asthma
FC: Fold change
GEO: Gene expression omnibus
GO: Gene ontology
ICS: Inhaled corticosteroids
KEGG: Kyoto encyclopedia of genes and genomes
MCC: Maximal clique centrality
MGA: Mixed granulocytic asthma
NA: Neutrophilic asthma
NEA: Non-eosinophilic asthma
PGA: Paucigranulocytic asthma
PPI: Protein–protein interaction network
ssGSEA: Single sample gene set enrichment analysis
TOM: Topological overlap matrix
WGCNA: Weighted gene co-expression network analysis
