# Supplementary material for: Weighted gene co-expression network analysis to identify key modules and hub genes associated with paucigranulocytic asthma
Source: BMC Pulm Med. 2021 Nov 2;21:343. doi: 10.1186/s12890-021-01711-3 (PMC8565058; doi:10.1186/s12890-021-01711-3)
Supplement: Supplementary file 1 — Additional file 1. Table S1: Baseline characteristics of the GSE45111 and GSE137268; Table S2 and S3; Validation of the hub genes; Figure S1: sample dendrogram and trait heatmap; Figure S2: Protein–protein interaction network analysis. [file 12890_2021_1711_MOESM1_ESM.docx]

**Supplemental Material**

**Weighted gene co‑expression network analysis to identify key modules and hub genes associated with paucigranulocytic asthma**

**Min Li^1,2^**^†^**, Wenye Zhu^3^**^†^**, Chu Wang^2^ , Zhen Yuanyuan^2^, Shibo Sun^2^, Yan Fang^2^, and Zhuang Luo^2*^**

^1^Department of Respiratory and Critical Care Medicine, West China Hospital, Sichuan University, Chengdu, China

^2^Department of Respiratory and Critical Care Medicine, First Affiliated Hospital of Kunming Medical University, Kunming, China

^3^Department of Pharmacy, First Affiliated Hospital of Kunming Medical University, Kunming, China

^†^Min Li and Wenye Zhu contributed equally to this work

***Correspondence**: Prof. Zhuang Luo, Department of Respiratory and Critical Care Medicine, First Affiliated Hospital of Kunming Medical University, Kunming 650000, P.R. China. Email: [huxitougao@yeah.net](mailto:huxitougao@yeah.net)

**Supplemental Tables**

Table S1 Baseline characteristics of GSE45111 and GSE137268

| Characteristics | GSE45111 | GSE137268 | *z*/χ^2^ | *P-*Value |
| --- | --- | --- | --- | --- |
| N | 47 | 54 | - | - |
| Platform | GPL670 | GPL670 |  |  |
| Source tissue | Induced sputum | Induced sputum |  |  |
| Age, years, median (Q1,Q3) | 60 (49, 67) | 62 (49, 68) | -0.198 | 0.843 |
| Gender, n(%) |  |  |  |  |
| Male | 20 (42.56) | 24 (44.44) | 0.036 | 0.849 |
| Female | 27 (57.44) | 30 (55.56) |  |  |
| Inflammation phenotype |  |  |  |  |
| PGA | 18 (38.3) | 16 (29.6) | 0.837 | 0.360 |
| Non-PGA | 29 (61.7) | 38 (70.4) |  |  |

PGA: Paucigranulocytic Asthma

Table S2 Differential expression analysis of the hub genes between the PGA and non-PGA in GSE 45111

| Hub genes | logFC | AveExpr | P.Value | adj.P.Value |
| --- | --- | --- | --- | --- |
| ADCY2 | -0.71 | 0.22 | 2.03E-05 | 6.94E-04 |
| CXCL1 | -0.77 | 0.05 | 2.45E-04 | 3.02E-03 |
| ADCY3 | 0.60 | 0.01 | 2.19E-03 | 0.01 |
| GPR109B | -0.92 | 0.01 | 8.01E-03 | 0.03 |
| GPR109A | -0.85 | 0.05 | 9.01E-03 | 0.04 |
| FPRL1 | -0.62 | -0.02 | 0.01 | 0.04 |

FC, fold change; AveExpr, Average expression

Table S3 Differential expression analysis of the hub genes between the PGA and non-PGA in GSE 137268

| Hub genes | logFC | AveExpr | P.Value | adj.P.Value |
| --- | --- | --- | --- | --- |
| ADCY2 | -0.69 | 0.27 | 4.20E-05 | 2.29E-04 |
| CXCL1 | -0.86 | 0.20 | 2.55E-04 | 2.61E-03 |
| FPRL1 | -0.73 | 0.08 | 3.36E-03 | 0.02 |
| GPR109B | -1.00 | 0.17 | 3.53E-03 | 0.02 |
| GPR109A | -0.92 | 0.19 | 3.96E-03 | 0.02 |
| ADCY3 | 0.58 | -0.07 | 5.29E-03 | 0.02 |

FC, fold change; AveExpr, Average expression

**Supplemental Figures**


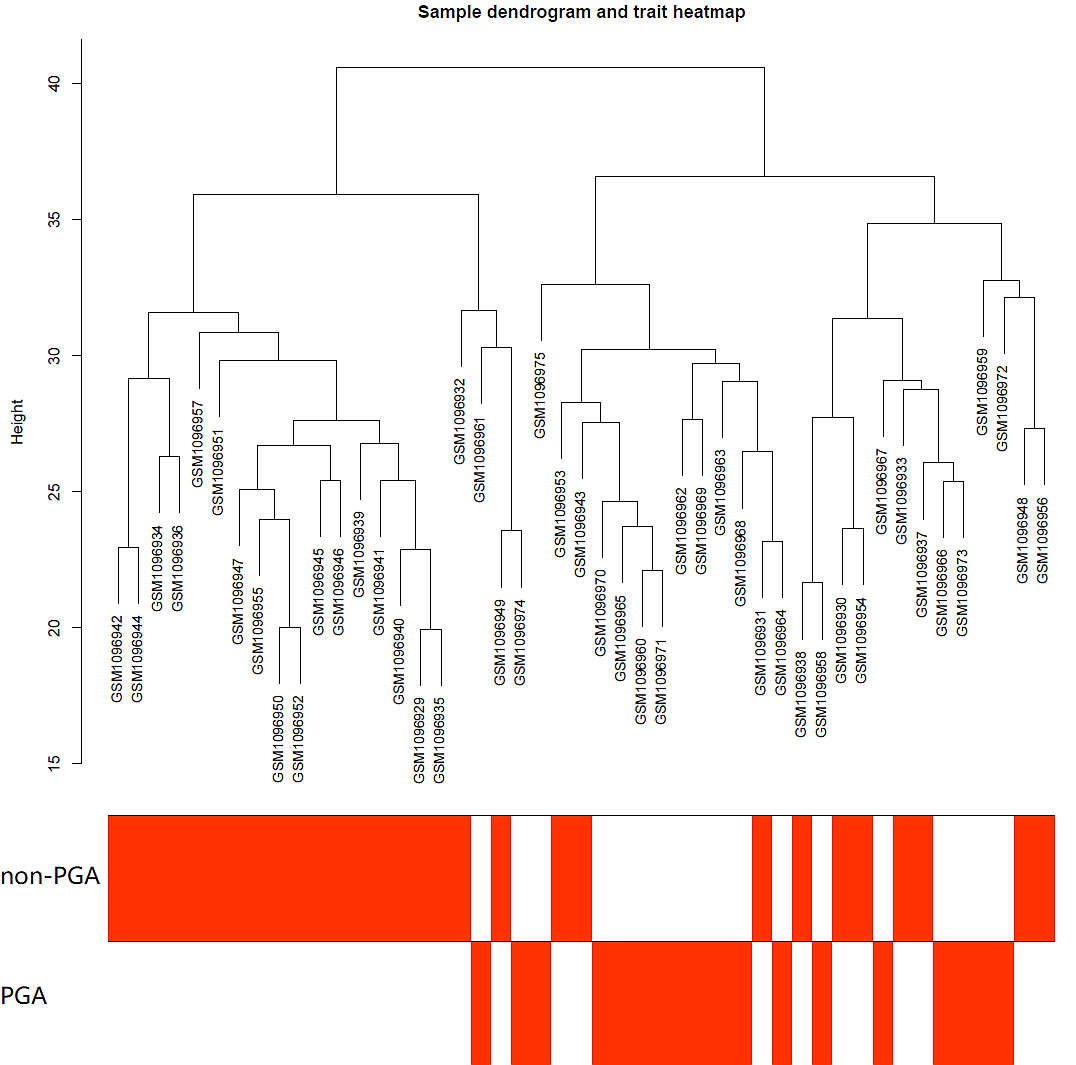


**Supplemental Figure S1.** Sample dendrogram and trait heatmap. The leaves of the tree correspond to the samples. Color bands represent the proportion to clinical traits (PGA or non-PGA)


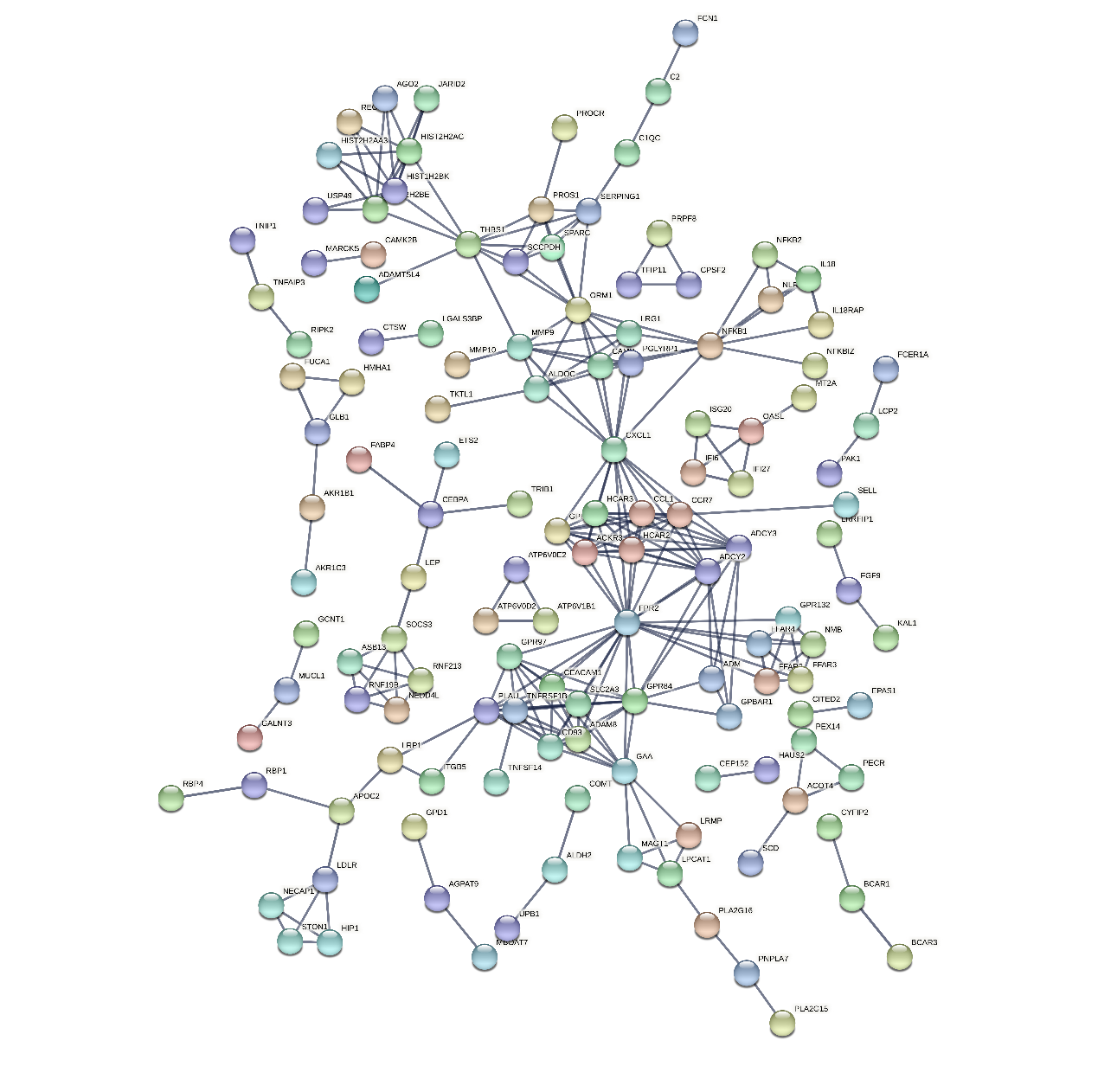
**Supplemental Figure S2**. Protein–protein interaction network analysis. Visualization of the network connections among the most highly connected genes within the overlapped genes.
